# Planetary health diet index and low muscle mass in adults aged 20–60 years: exploratory evaluation of a dairy-upweighted outcome-specific adaptation

**DOI:** 10.3389/fnut.2026.1782327

**Published:** 2026-07-15

**Authors:** Jin-jie He, Xu-lin Chen, Chao Ning, Qing-jun Wei

**Affiliations:** Department of Bone and Joint Diseases Surgery, The Second Affiliated Hospital of Guangxi Medical University, Nanning, Guangxi, China

**Keywords:** eat-lancet, low muscle mass, machine learning, multivariable logistic regression, restricted cubic spline

## Abstract

**Background:**

Increasing evidence indicates that the Planetary Health Diet Index (PHDI), proposed within the EAT-Lancet framework, not only supports environmental sustainability but also contributes to the prevention of various diseases. However, the rigorous restriction of animal-sourced proteins in the standard PHDI may overlook the heightened anabolic needs of aging populations. This study primarily aimed to evaluate the association between PHDI-US adherence and low muscle mass (LMM) and to compare this association with other commonly used dietary indices. As a secondary exploratory step, we examined whether an exploratory dairy-upweighted score, derived by multiplying the original PHDI-US total score by the observed dairy intake amount, might show a stronger association with LMM.

**Methods:**

Multivariable logistic regression and restricted cubic spline (RCS) models were used to evaluate the association between the Planetary Health Diet Index for the United States (PHDI-US) and low muscle mass (LMM). Random-forest feature-importance analysis was then used to identify the most influential dietary component within the PHDI-US and to derive a dairy-upweighted version of the score (PHDI-Dairy). We then examined the association between dairy intake and LMM in an independent Southern Chinese cohort.

**Results:**

In fully adjusted models, higher PHDI-US and Alternative Healthy Eating Index scores were associated with lower odds of LMM, whereas a higher Dietary Inflammatory Index score was associated with higher odds of LMM. Healthy Eating Index and Mediterranean Diet Index scores were not statistically significant in fully adjusted quartile analyses. In exploratory analyses, a dairy-upweighted score (PHDI-Dairy) showed a stronger inverse association with LMM than the original PHDI-US. In a separate hospital-based Southern Chinese cohort, higher dairy intake was associated with lower odds of LMM, providing supportive evidence for the dairy-related finding.

**Conclusion:**

PHDI-US was modestly associated with lower odds of LMM, and the exploratory dairy-upweighted score, PHDI-Dairy, showed a stronger inverse association with LMM.

## 1 Background

While sarcopenia is traditionally viewed as a geriatric syndrome, recent evidence suggests that its trajectory begins much earlier in life (1). Maximizing ‘peak muscle mass’ in early adulthood acts as a biological buffer against age-related decline (2). Therefore, identifying sustainable dietary patterns that support muscle maintenance in both younger and older adults is important for a life-course approach to sarcopenia prevention (3, 4). Within the current diagnostic pathway, low muscle mass (LMM) represents a central, relatively objective, and quantifiable component of the sarcopenia assessment continuum (5). Moreover, an increasing body of evidence indicates that even in the absence of a full sarcopenia diagnosis, isolated LMM is itself associated with adverse outcomes, including elevated risks of disability and all-cause mortality (6, 7). In addition, preventing muscle mass loss in early adulthood may help avert subsequent progression to overt sarcopenia later in life (8–10). Although sarcopenia is most commonly recognized in older adults, accumulating evidence supports a life-course perspective in which later-life muscle health is influenced by both age-related loss and the peak muscle reserve achieved earlier in adulthood. Therefore, identifying modifiable dietary patterns associated with low muscle mass before overt sarcopenia becomes more prevalent may be important for early prevention. Therefore, from the perspective of disease progression, we selected LMM as the primary study outcome to characterize an early adverse body-composition phenotype along the sarcopenia spectrum.

Low muscle mass (LMM) is generally defined using reduced appendicular lean mass assessed relative to height, weight, or body mass index (BMI), and constitutes one of the core components in the diagnosis of sarcopenia (11). The prevalence of LMM varies substantially according to the measurement methods and cut-off points applied. When definitions based solely on muscle quantity are used [e.g., appendicular lean mass (ALM)/weight, ALM/height^2^, or ALM/BMI], the pooled prevalence of low-muscle-mass-related phenotypes has been reported to reach 24–40% (12). Between 2017 and 2018, an estimated 28.7 million individuals, accounting for 15.9% of the U.S. population, were classified as having obesity with low lean muscle mass (OLLMM). Among older adults (≥60 years), 28.3% had OLLMM, and the prevalence reached 66.6% among Mexican-American women in this age group (13). According to the original European Working Group on Sarcopenia in Older People (EWGSOP) definition, pre-sarcopenia refers to isolated low muscle mass in the absence of impaired muscle strength or physical performance (14). In a sample of 29,947 adults aged 18–90 years, the overall prevalence of this phenotype was approximately 14.8%, and remained relatively stable over 7 years of follow-up. The highest prevalence was observed in individuals aged ≥80 years, whereas increasing trends were noted in young adults aged 18–39 years and in non-Hispanic Black participants (15). In a cohort of 1,211 community-dwelling older adults, the prevalence of low muscle mass defined using DXA-derived ALM/height^2^ was approximately 59.9% (16). Collectively, these findings indicate that LMM is highly prevalent in the general population. The pathogenesis of sarcopenia and LMM is multifactorial, involving multiple interrelated pathways, including chronic inflammation, impaired protein synthesis, insulin resistance, and mitochondrial dysfunction, among others (17). Among the various risk factors, lifestyle-particularly diet and physical activity-is regarded as the most modifiable component (3, 18). From a prevention perspective, dietary strategies that preserve muscle mass may provide an accessible approach to reducing the risk of low muscle mass, a central feature on the pathway toward sarcopenia (18).

In 2019, the Planetary Health Diet (PHD) framework proposed by the EAT-Lancet Commission emphasised both human health and environmental sustainability (19). This plant-forward dietary pattern, when combined with appropriate amounts of high-quality animal-source protein, may offer a promising nutritional strategy for supporting muscle health and maintaining muscle mass (19, 20). Plant-based foods provide antioxidants and can also contribute branched-chain amino acids, which may help reduce oxidative stress and support muscle metabolism (21, 22). In contrast, animal-source proteins generally provide a more complete essential amino acid profile and a higher leucine density, which may be advantageous for stimulating muscle protein synthesis (20, 23, 24). This balanced dietary pattern may help meet the nutritional requirements for muscle health while remaining aligned with environmental sustainability goals (19, 20).

However, whether the Planetary Health Diet Index for the United States (PHDI-US), designed to operationalize sustainable health goals, confers advantages in predicting low muscle mass remains unclear (25). Aging muscles often develop “anabolic resistance,” requiring higher doses of high-quality protein, particularly leucine-rich protein, to stimulate muscle protein synthesis (23, 26, 27). The standard EAT-Lancet framework limits dairy and red meat for environmental reasons (19), which creates a potential dilemma for preserving muscle mass in ageing populations. Therefore, a critical scientific question arises: Is it necessary to recalibrate the dairy component within PHDI to balance planetary health with the preservation of muscle mass? Accordingly, this study primarily aimed to evaluate the association between PHDI-US and low muscle mass, with other dietary indices included as comparators to contextualize the observed association. As a secondary exploratory analysis, we then used machine learning to identify influential dietary components within PHDI-US and to examine whether a refined version of the index might show a stronger association with LMM.

## 2 Methods

### 2.1 Study population

**Figure 1:**
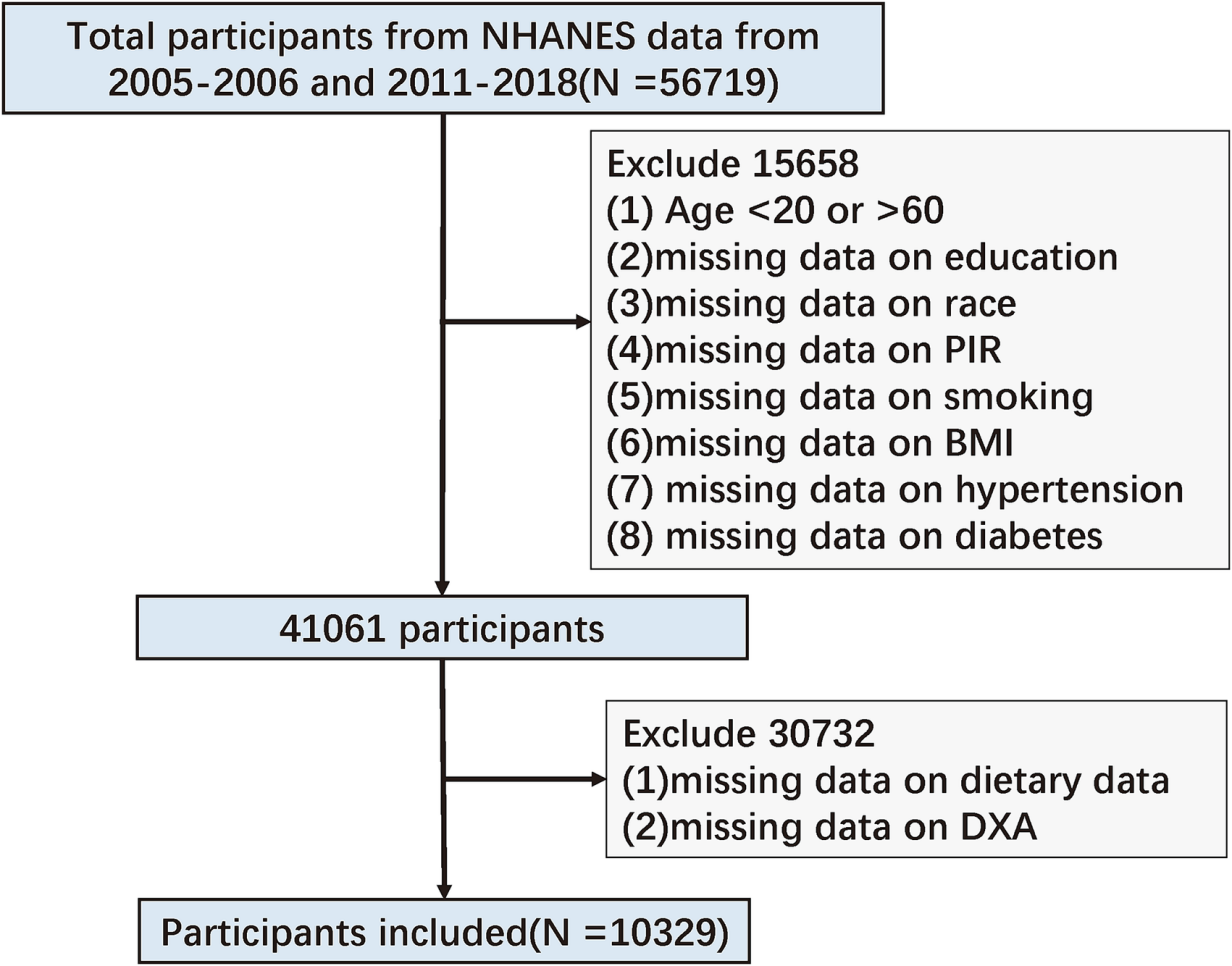
Flow chart of participant selection in the present study. The flow chart shows the selection process of eligible NHANES participants aged 20–60 years. Participants were excluded if they had missing dietary data, missing DXA-derived appendicular lean mass data, or missing key covariates. The final analytic sample included adults with complete information on dietary indices, low muscle mass status, and covariates.

This study used data from the National Health and Nutrition Examination Survey (NHANES) (28), and included 10,329 adults aged 20–60 years with complete dietary, covariate, and DXA-derived appendicular lean mass data. All participants provided written informed consent, and the study protocol strictly followed the ethical guidelines of the Declaration of Helsinki and was approved by the Research Ethics Review Board of the National Center for Health Statistics (NCHS) (29). The study population covered groups of different genders, ages, races, and socioeconomic statuses, and was representative of the national population (28). Throughout the research process, NHANES data usage guidelines were strictly followed to ensure compliance with ethical requirements in medical research (29). The inclusion years of this study were 2005–2006 and 2011–2018, covering a total of five cycles. This is because complete dietary diet index and dual-energy X-ray absorptiometry (DXA) data were available in these five cycles. Strict exclusion criteria were applied to ensure data quality: (1) individuals younger than 20 years; (2) those missing key covariates (including demographic characteristics and socioeconomic status); (3) those missing dietary data; and (4) those missing DXA-derived appendicular lean mass index data. After applying the above selection criteria, a final study cohort of 10,329 eligible subjects with complete data was included. All data can be accessed via the NCHS website.^1^ (see Figure 1).

### 2.2 Dietary assessment and index calculation

Dietary intake data of NHANES participants were obtained through computer-assisted face-to-face interviews conducted at mobile examination centres (30). For dietary index calculation, we used the processed two-day dietary recall information available within the NHANES-based dietary index framework, rather than relying only on the first recall day. Total food, beverage intake (excluding water), and nutrient variables used for index construction were derived from these processed dietary data (31). In addition to PHDI-US, we included the Alternative Healthy Eating Index 2010(AHEI-2010), Healthy Eating Index 2015(HEI), Mediterranean Diet Index (MEDI), and Dietary Inflammatory Index (DII) as comparator indices because they are established measures of overall diet quality, dietary guideline adherence, Mediterranean-style dietary adherence, or dietary inflammatory potential, and have previously been linked to sarcopenia-related or muscle-related outcomes. These indices therefore served as relevant contextual benchmarks for interpreting the observed association for PHDI-US. The study used NHANES data from 2005–2006 and 2011–2018 to assess each participant’s total food and beverage intake (excluding water) and nutrient consumption. The USDA Food Patterns Equivalents Database (FPED) was used to calculate the Food Patterns Equivalents (FPE) for NHANES participants from 2005 and 2018.^2^ The PHDI-US was constructed and scored in accordance with the original EAT-Lancet Commission framework published in 2019, which defines the dietary targets underlying the PHDI scoring system (19). The PHDI-US uses a multidomain scoring structure to summarize different aspects of dietary intake, including adequacy, optimality, ratio, and moderation components (32). Detailed component-specific scoring criteria, intake ranges, and maximum scores are provided in Supplementary Table S1. Among them, nuts, legumes, etc., are classified as adequacy components; zero intake reduces diet quality, while reaching the reference value poses no harm. Optimum components include seafood and substitutes, tubers and starchy vegetables, dairy, and unsaturated oils. Two ratio components-dark green vegetables and red/orange vegetables-are capped at a maximum score of 5 to avoid overestimation. Moderation components include eggs, red and processed meat, poultry, saturated fats, and added sugars. Each section has a maximum score of 10 or 5 points, with a total score range of 0–150. PHDI demonstrates validity and reliability. A higher PHDI-US score indicates greater adherence to the Planetary Health dietary recommendations (33). Dietary Index is a standardized, user-friendly, and validated informatics tool specifically designed for calculating dietary indices. Its validation documentation, source code, and detailed user tutorials are publicly available on the GitHub platform^3^ (34). This R package can calculate multiple dietary indices, including PHDI-US, the Dietary Inflammatory Index (DII), the Alternative Healthy Eating Index 2010(AHEI-2010), the Healthy Eating Index 2015(HEI-2015), and the Mediterranean Diet Index (MEDI). This approach was consistent with the standardized implementation of NHANES-based dietary index construction and avoided describing the exposure assessment as based only on Day 1 recall.

### 2.3 Construction of the PHDI-dairy

To develop an exploratory outcome-specific adaptation of the U.S. Planetary Health Diet Index (PHDI-US) for low muscle mass, machine learning-based feature-importance analyses were conducted using a random forest model with recursive feature elimination. Mean decrease in accuracy and Gini importance were used to rank the relative contributions of the 16 PHDI components. Dairy intake consistently showed the highest feature importance. Dairy was identified as the highest-importance component. We therefore created an exploratory dairy-upweighted score (PHDI-Dairy) by multiplying the original PHDI-US total score by the observed dairy intake amount. PHDI-Dairy should be interpreted as an exploratory outcome-specific adaptation of PHDI-US for LMM, constructed by multiplying the original PHDI-US total score by the observed dairy intake amount. Higher scores indicate greater adherence to the PHDI-US framework with increased emphasis on dairy intake in the context of this specific outcome. Reweighting the dairy component was intended only as an exploratory, outcome-specific analytical adaptation for low muscle mass and should not be interpreted as redefining or extending the original planetary health diet framework itself.

### 2.4 LMM definition

In this study, dual-energy X-ray absorptiometry (DXA) was used to assess appendicular lean mass and to define LMM. The appendicular lean mass index was calculated by normalizing appendicular lean mass to height squared (kg/m^2^). Low muscle mass was defined using sex-specific cut-offs of 7.0 kg/m^2^ for men and 5.5 kg/m^2^ for women (5).

### 2.5 Variables

The sociodemographic characteristics in this study were collected through standardized questionnaires and face-to-face interviews, covering age (≥20 years, ≤60 years), gender (male/female), race (Mexican American/Non-Hispanic White/Non-Hispanic Black/Other Race-Including Multi-Racial/Other Hispanic), education level (Below high school/High school/Above high school), family poverty income ratio (PIR, Below poverty threshold/Above poverty threshold), and diabetes status. Behavioural indicators included smoking status (non-smoker/smoker) and alcohol consumption status (drinker/non-drinker). Physical examination indicators comprised BMI (weight kg/height m^2^) and blood pressure status. A BMI of 25 ≤ BMI < 30 kg/m^2^ was defined as overweight, and a BMI of ≥30 kg/m^2^ was defined as obesity. Blood pressure measurements were required to be the average of three standardised measurements (taken at 30-s intervals). Diabetes was defined as the presence of any of the following: self-reported physician diagnosis of diabetes, use of glucose-lowering medication, or meeting the laboratory diagnostic criteria for diabetes. To control for time effects, subgroup analyses were conducted by survey period (2005–2006/2011–2012 vs. 2013–2018), based on the available NHANES cycles included in this study. To explore potential heterogeneity by sex and female age group, stratified analyses were performed by sex and by female age category (20–45 years and 46–60 years). These analyses were intended to examine subgroup variation rather than to directly infer hormonal mechanisms.

### 2.6 Association between dairy intake and low muscle mass in the Southern Chinese cohort

This retrospective observational analysis used data from a hospital-based Southern Chinese sample at The Second Affiliated Hospital of Guangxi Medical University, Nanning, Guangxi, China, collected between January 2025 and July 2025. No special recruitment was performed for the present study. Instead, participants were retrospectively identified from hospital examination records among patients with available DXA data. Thus, the sampling frame was hospital-based and was not intended to represent the general Southern Chinese population.

Eligible participants were adults aged 20–60 years who had complete DXA measurements, complete dairy-intake questionnaire data, and complete covariate information. Exclusion criteria included missing key variables, pregnancy, uninterpretable DXA findings, duplicate records, and implausible dietary values. Dairy intake was assessed by direct telephone interview with the participant, and two-day dairy intake data were collected. Intake was then quantified according to the EAT-Lancet framework using an R package-based approach. In the Southern Chinese cohort, 218 participants were included: 107 with LMM and 111 without LMM. Because this dietary assessment method differed substantially from the NHANES processed two-day dietary recall approach, and because the Southern Chinese sample was hospital-based rather than nationally representative, this analysis was interpreted as supportive evidence for the dairy-related association rather than as formal external validation of the full PHDI-Dairy composite score. The fully adjusted model in this cohort included only the covariates actually collected: age, sex, BMI, smoking status, and hypertension. The study was approved by the Ethical Review Committee of The Second Affiliated Hospital of Guangxi Medical University (approval number: 2025-KYL [001]).

### 2.7 Statistical analysis

Statistical analyses incorporated sample weights, stratification, and clustering factors from the NHANES multistage probability sampling design for inferential analyses. Analyses were performed using R software version 4.4.1. Table 1 presents unweighted counts and percentages to describe the analytic sample, whereas between-group comparisons accounted for the NHANES complex survey design using appropriate survey-weighted procedures. For between-group comparisons, continuous variables were assessed using weighted Student’s t-tests, and categorical variables were evaluated via Rao-Scott chi-square tests.

**Table 1:** Baseline characteristics of the analytic NHANES sample by LMM status.

Variable	Non-LMM	With LMM	*p*-value
Gender			0.43
Female	4,172.00 (44.06%)	367.00 (42.67%)	
Male	5,297.00 (55.94%)	493.00 (57.33%)	
Age			<0.001
20–45 years	6,452.00 (68.14%)	440.00 (51.16%)	
46–60 years	3,017.00 (31.86%)	420.00 (48.84%)	
BMI			<0.001
<18.5	178.00 (1.88%)	3.00 (0.35%)	
18.5 to <25	3,085.00 (32.58%)	69.00 (8.02%)	
25 to <30	3,063.00 (32.35%)	212.00 (24.65%)	
> = 30	3,143.00 (33.19%)	576.00 (66.98%)	
Race			<0.001
Mexican American	1,322.00 (13.96%)	311.00 (36.16%)	
Other Hispanic	784.00 (8.28%)	124.00 (14.42%)	
Non-Hispanic White	3,808.00 (40.22%)	252.00 (29.30%)	
Non-Hispanic Black	2,067.00 (21.83%)	53.00 (6.16%)	
Other Race - Including Multi-Racia	1,488.00 (15.71%)	120.00 (13.95%)	
PIR			<0.001
Below poverty threshold	4,130.00 (43.62%)	499.00 (58.02%)	
Above poverty threshold	5,339.00 (56.38%)	361.00 (41.98%)	
Education			<0.001
Below high school	1,500.00 (15.84%)	266.00 (30.93%)	
High school	2,035.00 (21.49%)	226.00 (26.28%)	
Above high school	5,934.00 (62.67%)	368.00 (42.79%)	
Smoking			<0.001
NO	7,417.00 (78.33%)	604.00 (70.23%)	
YES	2,052.00 (21.67%)	256.00 (29.77%)	
Hypertension			0.030
NO	5,598.00 (59.12%)	541.00 (62.91%)	
YES	3,871.00 (40.88%)	319.00 (37.09%)	
Diabetes			<0.001
NO	8,577.00 (90.58%)	670.00 (77.91%)	
YES	892.00 (9.42%)	190.00 (22.09%)	

Data are expressed as frequency (percentage). PIR, ratio of family income to poverty; BMI, body mass index.

Participants were categorised into quartiles based on PHDI-US scores, with the lowest quartile (Q1) as the reference. To examine the association between PHDI-US and LMM, three progressively adjusted multivariable logistic regression models were constructed: Model 1 included no covariates; Model 2 adjusted for sex and age; and Model 3 further incorporated age, sex, race/ethnicity, poverty-to-income ratio, education level, BMI, smoking status, hypertension, and diabetes. Odds ratios (ORs) with 95% confidence intervals (CIs) were calculated. Restricted cubic spline (RCS) analysis was employed to assess nonlinear relationships between dietary indices, including PHDI-US and AHEI-2010, and LMM. To explore whether increasing the influence of the most relevant dietary component could improve the applicability of the U.S. Planetary Health Diet Index (PHDI-US) for low muscle mass, we first used a random forest model to rank the relative importance of the 16 PHDI-US components. Dairy intake consistently emerged as the most important component. We therefore constructed an exploratory modified index (PHDI-Dairy) by multiplying the original PHDI-US total score by the observed dairy intake amount. This procedure was intended as a simple proof-of-concept modification strategy rather than a formal optimization of component weights. Accordingly, PHDI-Dairy should be interpreted as an exploratory index used to assess whether emphasising dairy could strengthen the association between the original PHDI framework and low muscle mass. Multivariable RCS was also applied for stratified analyses by year, sex, and age. A two-sided *p*-value <0.05 was considered statistically significant. Given the physiological differences in muscle metabolism across adulthood, we performed age-stratified analyses using the same categories throughout the manuscript (20–45 and 46–60 years) to investigate whether the association between diet and low muscle mass varied by age group.

In addition, a hospital-based Southern Chinese cohort was analysed to assess the association between dairy intake and LMM. Multivariable logistic regression models were applied to evaluate the association between dairy intake and LMM, with progressive adjustment for age, sex, BMI, smoking, and hypertension. Restricted cubic spline analysis was further employed to assess potential nonlinear relationships. We performed repeated 5-fold cross-validation within NHANES. The dairy weight was derived only in the training folds and then applied unchanged to the corresponding validation folds. Associations with low muscle mass were re-evaluated in the validation data using multivariable logistic regression.

## 3 Results

### 3.1 Baseline characteristics of the study sample

A total of 10,329 participants were included in this study, including 9,469 in the non-LMM group and 860 in the LMM group. As shown in Table 1, sex distribution was comparable between the two groups (*p* = 0.43). In contrast, significant differences were observed in age, BMI, race, PIR, education, smoking status, hypertension, and diabetes (all *p* < 0.05). Compared with the non-LMM group, the LMM group had a higher proportion of participants aged 46–60 years (48.84% vs. 31.86%), a markedly higher proportion with BMI > 30 kg/m^2^ (66.98% vs. 33.19%), and a higher proportion of Mexican American participants (36.16% vs. 13.96%). Participants with LMM were also more likely to be below the poverty threshold (58.02% vs. 43.62%), to have lower educational attainment, to be smokers (29.77% vs. 21.67%), and to have diabetes (22.09% vs. 9.42%). In addition, the prevalence of hypertension was lower in the LMM group than in the non-LMM group (37.09% vs. 40.88%, *p* = 0.030). More details can be seen in Table 1.

### 3.2 The relationship between PHDI-US, AHEI, DII, HEI, MEDI, and LMM

**Figure 2:**
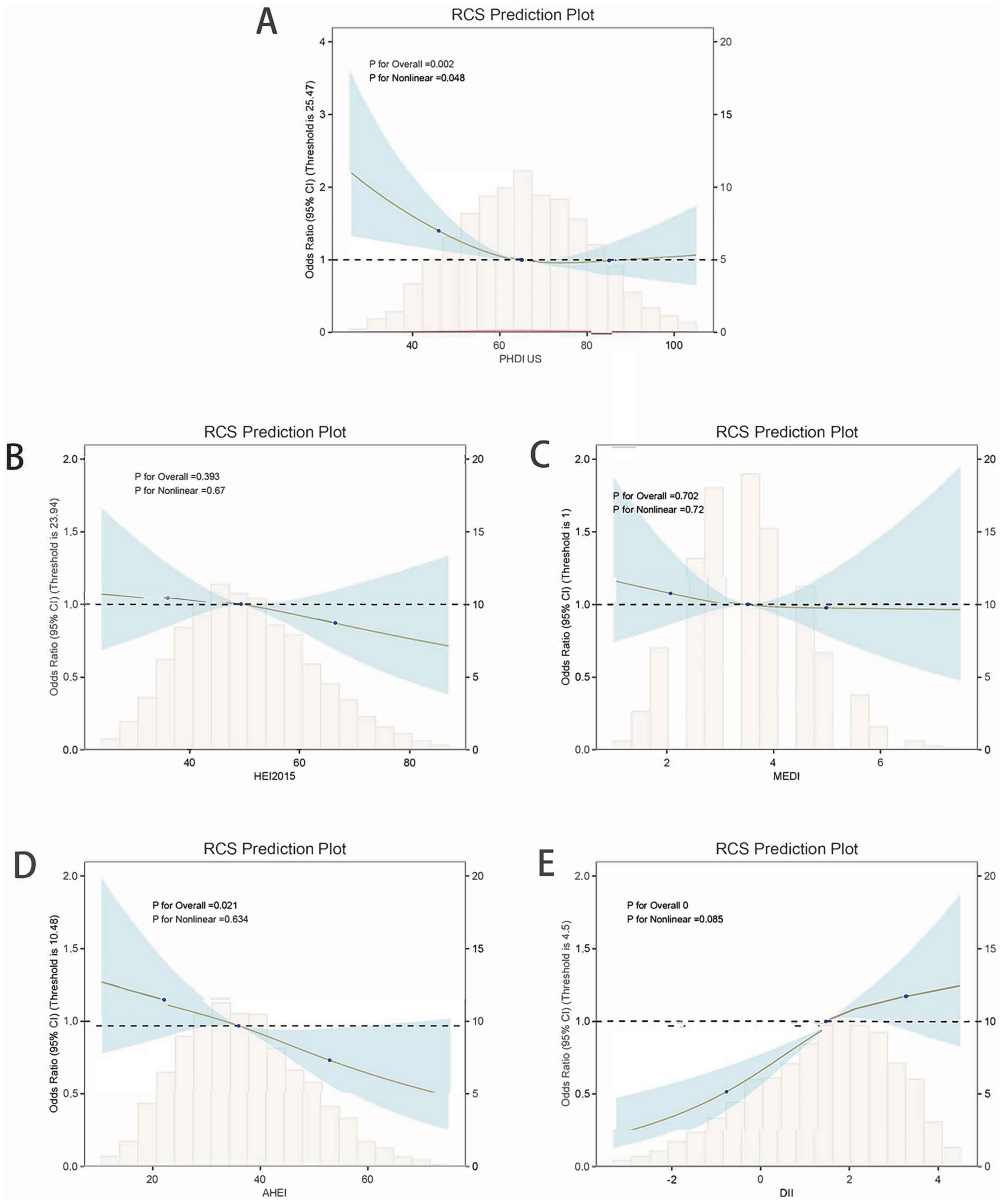
Restricted cubic spline analysis of the associations between dietary quality indices and low muscle mass. Panel **(A)** shows PHDI-US; Panel **(B)** shows HEI-2015; Panel **(C)** shows MEDI; Panel **(D)** shows AHEI; Panel **(E)** shows DII. Restricted cubic spline models were used to evaluate the dose–response associations of PHDI-US, HEI-2015, MEDI, AHEI, and DII with low muscle mass. Odds ratios and 95% confidence intervals are shown across the distribution of each dietary index. Models were adjusted for age, sex, race/ethnicity, PIR, education, BMI, smoking status, hypertension, and diabetes. PHDI-US, Planetary Health Diet Index for the United States; HEI-2015, Healthy Eating Index 2015; MEDI, Mediterranean Diet Index; AHEI, Alternative Healthy Eating Index; DII, Dietary Inflammatory Index; LMM, low muscle mass; OR, odds ratio; CI, confidence interval.

Restricted cubic spline (RCS) analysis (Figure 2) showed an inverse association of AHEI with LMM and a more modest inverse association of PHDI-US with LMM. In contrast, DII showed a positive association with LMM. No statistically significant overall associations were observed for HEI or MEDI in the spline analyses. Logistic regression analysis (Table 2) showed that individuals in the highest quartile of AHEI (Q4) had significantly lower odds of LMM (Q4 OR = 0.67, 95% CI: 0.51–0.87, *p* < 0.01) after full adjustment (Model 3). However, the inverse association between PHDI-US and LMM was more modest; in the fully adjusted quartile model, participants in Q4 had lower odds of LMM than those in Q1 (OR = 0.73, 95% CI: 0.57–0.95; *p* = 0.02). DII was significantly positively associated with LMM (Q4 OR = 1.94, 95% CI: 1.49–2.53, *p* < 0.01). In Model 3, HEI and MEDI showed no statistically significant associations with LMM.

**Table 2:** Associations between dietary quality indices and LMM by quartiles.

Dietary quality indices	Model 1 OR (95%CI)	*p*-value	Model 2 OR (95%CI)	*p*-value	Model 3 OR (95%CI)	*p*-value
PHDI-US						
Quartile 1	Reference	–	Reference	–	Reference	–
Quartile 2	0.91 (0.72–1.14)	0.41	0.89 (0.71–1.13)	0.32	0.84 (0.66–1.09)	0.20
Quartile 3	0.97 (0.77–1.23)	0.81	0.94 (0.75–1.19)	0.60	0.86 (0.67–1.11)	0.25
Quartile 4	0.91 (0.72–1.14)	0.40	0.87 (0.69–1.10)	0.24	0.73 (0.57–0.95)	0.02
P for trend		0.54		0.32		<0.01
AHEI						
Quartile 1	Reference	–	Reference	–	Reference	–
Quartile 2	1.17 (0.94–1.47)	0.15	1.10 (0.88–1.37)	0.42	1.06 (0.84–1.34)	0.62
Quartile 3	0.93 (0.73–1.17)	0.51	0.82 (0.65–1.03)	0.09	0.74 (0.57–0.94)	0.02
Quartile 4	0.72 (0.56–0.92)	<0.01	0.60 (0.47–0.77)	<0.01	0.67 (0.51–0.87)	<0.01
P for trend		<0.01		<0.01		<0.01
DII						
Quartile 1	Reference	–	Reference	–	Reference	–
Quartile 2	1.38 (1.07–1.80)	0.02	1.41 (1.09–1.83)	0.01	1.31 (1.00–1.72)	0.05
Quartile 3	1.77 (1.38–2.28)	<0.01	1.88 (1.47–2.43)	<0.01	1.90 (1.46–2.47)	<0.01
Quartile 4	1.82 (1.43–2.35)	<0.01	1.96 (1.53–2.53)	<0.01	1.94 (1.49–2.53)	<0.01
P for trend		<0.01		<0.01		<0.01
HEI						
Quartile 1	Reference	–	Reference	–	Reference	–
Quartile 2	1.00 (0.79–1.26)	0.99	0.94 (0.74–1.19)	0.60	0.95 (0.74–1.21)	0.64
Quartile 3	1.04 (0.82–1.31)	0.76	0.96 (0.76–1.21)	0.73	0.92 (0.72–1.18)	0.51
Quartile 4	0.91 (0.72–1.15)	0.43	0.78 (0.61–0.99)	0.04	0.79 (0.61–1.02)	0.07
P for trend		0.52		0.06		0.08
MEDI						
Quartile 1	Reference	–	Reference	–	Reference	–
Quartile 2	1.03 (0.82–1.29)	0.8	0.98 (0.78–1.23)	0.88	0.92 (0.72–1.16)	0.48
Quartile 3	1.01 (0.80–1.29)	0.91	0.94 (0.73–1.20)	0.64	0.84 (0.64–1.08)	0.18
Quartile 4	0.90 (0.73–1.12)	0.38	0.84 (0.67–1.03)	0.10	0.81 (0.64–1.02)	0.08
P for trend		0.43		0.11		0.05

Model 1, No covariates were adjusted; Model 2, Adjusted for gender and age; Model 3 was adjusted for age, sex, race/ethnicity, PIR, education, BMI, smoking status, hypertension, and diabetes. PHDI-US, Planetary Health Diet Index for the United States; AHEI, Alternative Healthy Eating Index; DII, Dietary Inflammatory Index; HEI, Healthy Eating Index; MEDI, Mediterranean Diet Index; OR, odds ratio; CI, confidence interval.

### 3.3 Strengthening the PHDI-dairy association via targeted dairy component recalibration, repeated 5-fold cross-validation

**Figure 3:**
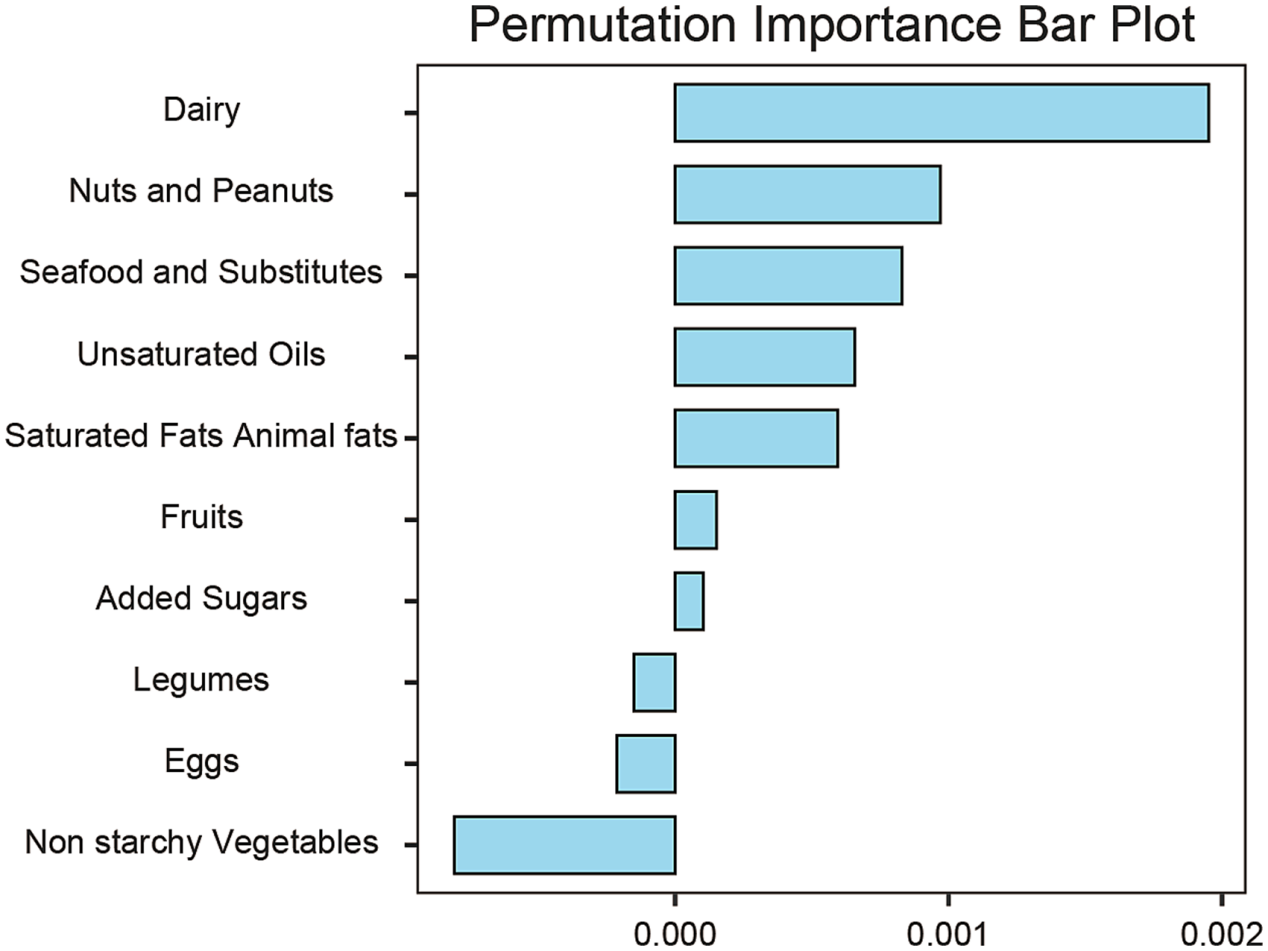
Relative contribution of PHDI dietary components to LMM risk prediction based on random forest permutation: Top 10 PHDI-US components by permutation importance. Random forest feature-importance analysis was used to rank the contribution of PHDI-US components to low muscle mass prediction. The figure presents the top 10 PHDI-US components ranked by permutation importance. Higher importance values indicate greater contribution to model prediction. PHDI-US, Planetary Health Diet Index for the United States; LMM, low muscle mass.

**Figure 4:**
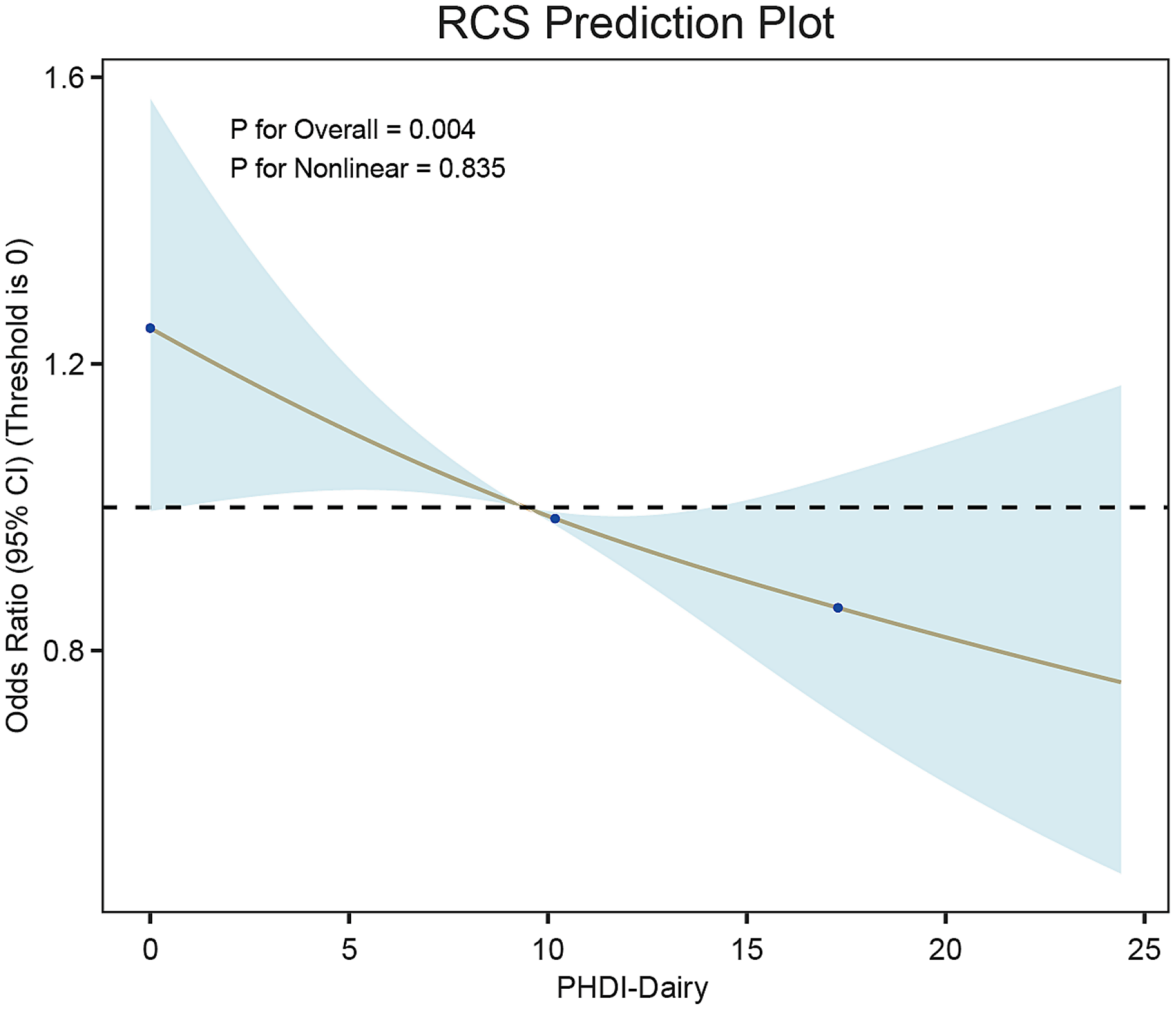
Association between PHDI-Dairy and LMM. Restricted cubic spline analysis was used to examine the dose–response association between PHDI-Dairy and low muscle mass. Odds ratios and 95% confidence intervals are shown across the distribution of PHDI-Dairy. The model was adjusted for age, sex, race/ethnicity, PIR, education, BMI, smoking status, hypertension, and diabetes. PHDI-Dairy was constructed by multiplying the original PHDI-US total score by observed dairy intake. PHDI-US, Planetary Health Diet Index for the United States; LMM, low muscle mass; OR, odds ratio; CI, confidence interval.

This study conducted a feature-importance analysis of the 16 dietary components of the PHDI using a random forest model. The machine learning analysis (Figure 3) indicated that dairy had the highest feature importance among all PHDI components. Based on this finding, we constructed an exploratory dairy-upweighted score (PHDI-Dairy) by multiplying the original PHDI-US total score by the observed dairy intake amount. After stepwise adjustment for confounding factors, PHDI-Dairy showed a significant inverse association with LMM (Table 3). In the fully adjusted model (Model 3), individuals in the highest quartile of PHDI-Dairy (Q4) had lower odds of LMM (OR = 0.68, 95% CI: 0.55–0.84, *p* < 0.01), with a significant trend across quartiles (*P* for trend < 0.01). RCS analysis (Figure 4) showed a continuously decreasing dose–response curve without an apparent plateau within the observed range, and the overall association was statistically significant (*P* for overall < 0.05).

**Table 3:** Association between PHDI-Dairy and low muscle mass (LMM).

PHDI-Dairy	Model 1 OR (95%CI)	*p*-value	Model 2 OR (95%CI)	*p*-value	Model 3 OR (95%CI)	*p*-value
Low	Reference	–	Reference	–	Reference	–
High	0.98 (0.97–0.99)	<0.01	0.98 (0.97–0.99)	0.02	0.97 (0.96–0.99)	<0.01
Interquartile						
Quartile 1	Reference	–	Reference	–	Reference	–
Quartile 2	0.91 (0.76–1.10)	0.36	0.93 (0.77–1.13)	0.49	0.90 (0.73–1.11)	0.34
Quartile 3	0.78 (0.65–0.96)	0.02	0.81 (0.66–0.99)	0.03	0.81 (0.65–1.00)	0.05
Quartile 4	0.78 (0.64–0.95)	0.01	0.79 (0.64–0.96)	0.02	0.68 (0.55–0.84)	<0.01
P for trend		<0.01		<0.01		<0.01

Model 1, No covariates were adjusted; Model 2, Adjusted for gender and age; Model 3, adjusted for age, sex, race/ethnicity, PIR, education, BMI, smoking status, hypertension, and diabetes. OR, Odds Ratio; CI, Confidence Interval.

In repeated 5-fold cross-validation, the dairy-upweighted score derived in the training folds showed a consistent inverse direction of association with low muscle mass in the held-out validation folds. The association was weaker than that observed in the full analytical sample, but the direction was consistent. Compared with the original PHDI-US, PHDI-Dairy showed more favorable validation metrics, with a lower mean OR for LMM per 1-SD increase (0.92 vs. 0.97) and a lower mean OR for LMM for Q4 versus Q1 (0.66 vs. 0.81). PHDI-Dairy also showed better discrimination than PHDI-US, with a mean validation AUROC of 0.69 versus 0.63. These findings suggest that the apparent advantage of PHDI-Dairy was partly retained in internal validation. However, the weaker effect size in validation data indicates that the full-sample estimates may be somewhat overestimated. Detailed results are presented in Table 4.

**Table 4:** Association stability and discrimination of PHDI-US and PHDI-Dairy for low muscle mass in repeated 5-fold cross-validation.

Index	Mean validation OR per SD (95% CI)	Mean validation OR, Q4 vs. Q1 (95% CI)	Mean validation AUROC
PHDI-US	0.97 (0.96–0.99)	0.81 (0.76–0.89)	0.63
PHDI-Dairy	0.92 (0.88–0.95)	0.66 (0.54–0.82)	0.69

ORs are presented with 95% CIs. OR per SD indicates the association with low muscle mass per 1-standard-deviation increase in the dietary index. Q4 vs Q1 compares the highest with the lowest quartile. AUROC indicates the area under the receiver operating characteristic curve. PHDI-US, Planetary Health Diet Index for the United States; PHDI-Dairy, dairy-upweighted PHDI-US; OR, odds ratio; CI, confidence interval; AUROC, area under the receiver operating characteristic curve.

### 3.4 Stratified analysis

**Figure 5:**
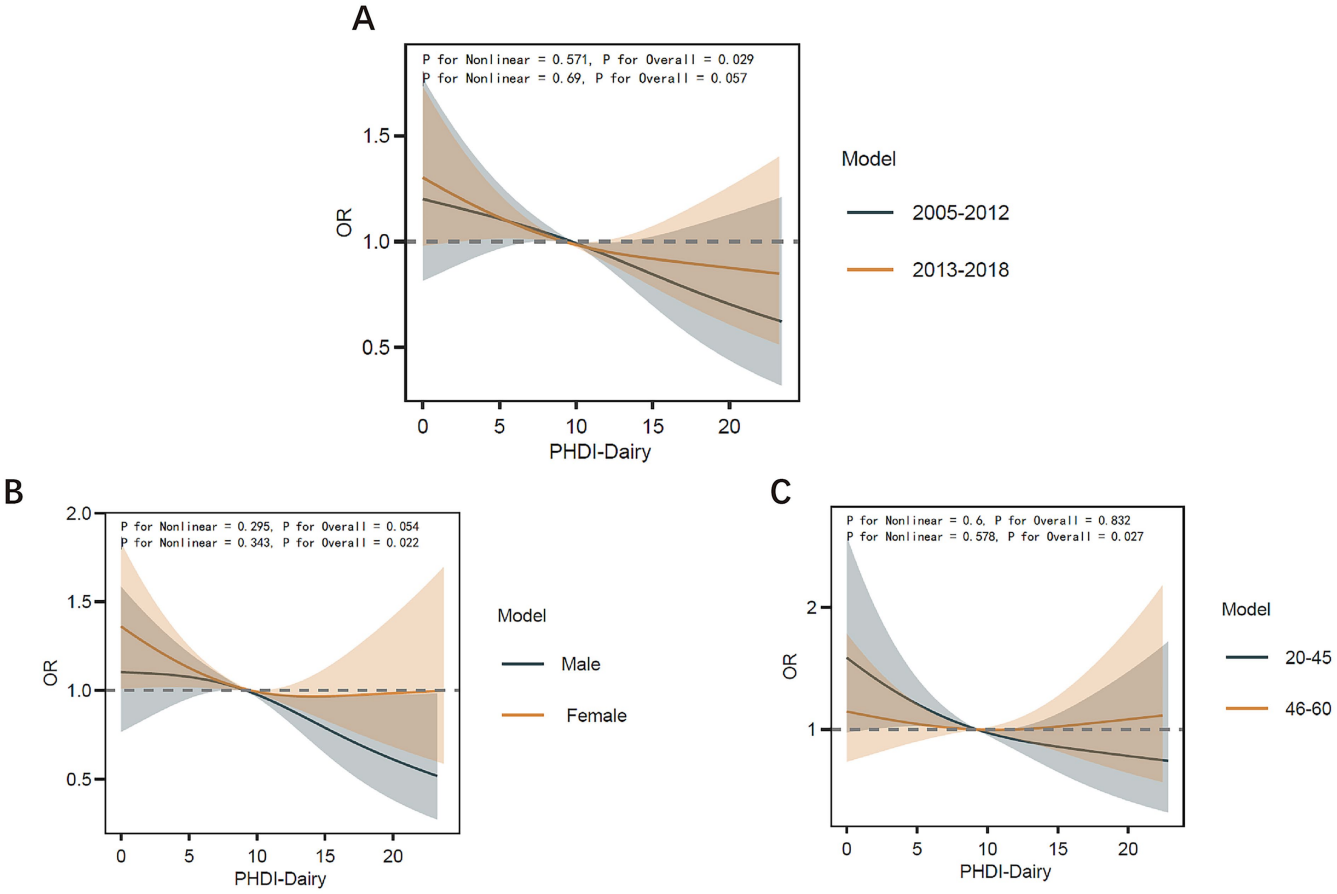
Subgroup restricted cubic spline analyses of the association between PHDI-Dairy and low muscle mass. Restricted cubic spline analyses were conducted to evaluate the association between PHDI-Dairy and low muscle mass across selected subgroups. Panel **(A)** shows results stratified by NHANES survey period; Panel **(B)** shows results stratified by sex; Panel **(C)** shows results stratified by female age group. Models were adjusted for age, sex, race/ethnicity, PIR, education, BMI, smoking status, hypertension, and diabetes, except for the corresponding stratification variable where appropriate. PHDI-Dairy was constructed by multiplying the original PHDI-US total score by observed dairy intake. PHDI-US, Planetary Health Diet Index for the United States; LMM, low muscle mass; OR, odds ratio; CI, confidence interval.

Multivariable restricted cubic spline (RCS) analysis showed that PHDI-Dairy was significantly inversely associated with LMM during 2005–2012 (P for overall < 0.05), whereas this association was not statistically significant during 2013–2018 (P for overall > 0.05). Subgroup analysis by sex showed a significant overall inverse association between PHDI-Dairy and LMM in women (P for overall < 0.05), although the curve appeared to attenuate at higher adherence levels. No statistically significant overall association was observed between PHDI-Dairy and LMM in men (P for overall = 0.054). Further age-subgroup analysis in women showed a significant inverse association between PHDI-Dairy and LMM in individuals aged 46–60 years (P for overall < 0.05), whereas no statistically significant association was observed in the 20–45 years age group (P for overall > 0.05) (Figure 5).

### 3.5 Cross-sectional association between dairy intake and low muscle mass in a Southern Chinese population

**Figure 6:**
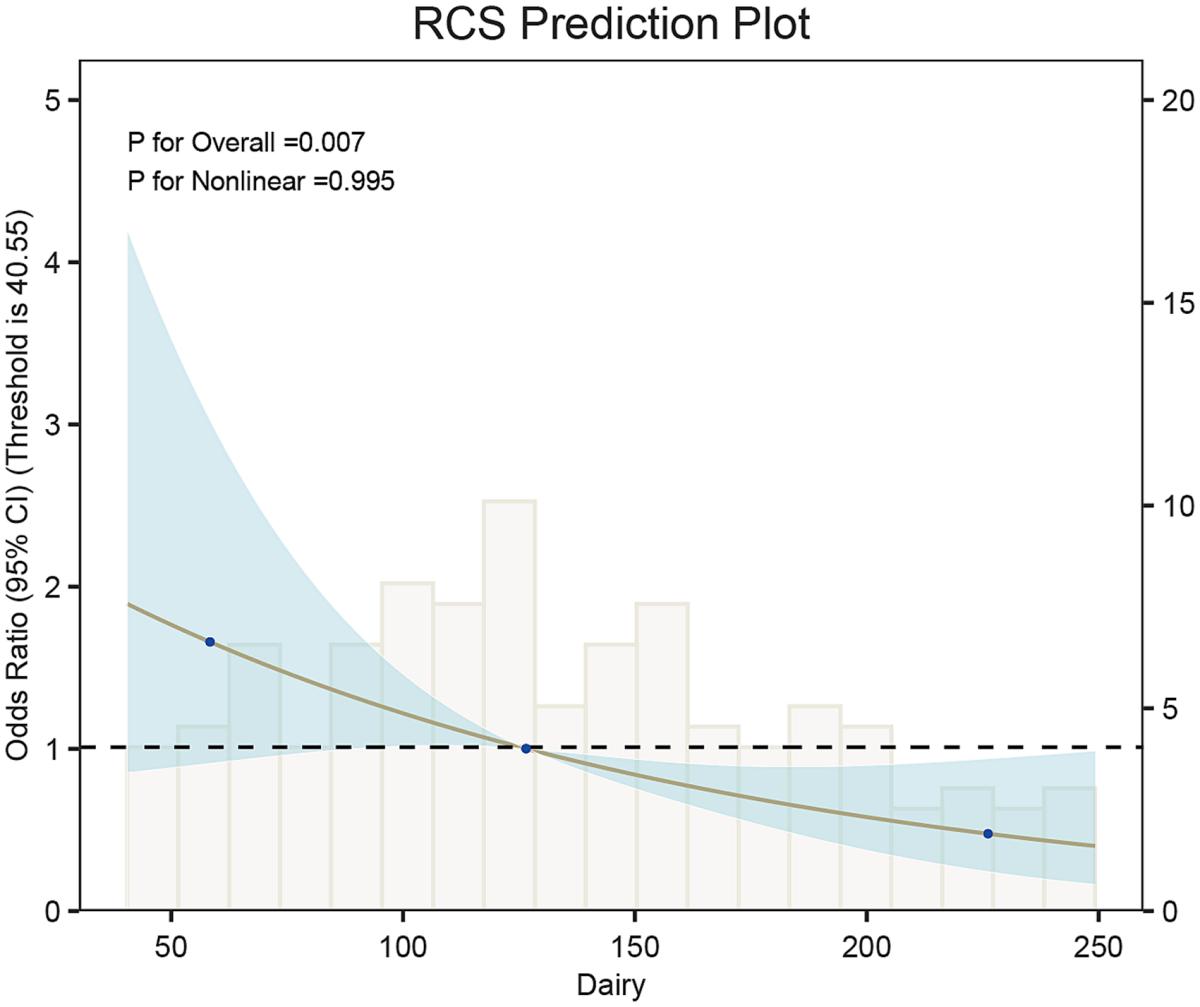
Association between dairy intake and low muscle mass in the Southern Chinese cohort. Restricted cubic spline analysis was used to examine the dose–response association between dairy intake and low muscle mass in the hospital-based Southern Chinese cohort. Odds ratios and 95% confidence intervals are shown across the distribution of dairy intake. The fully adjusted model included age, sex, BMI, smoking status, and hypertension. LMM, low muscle mass; OR, odds ratio; CI, confidence interval; BMI, body mass index.

In a hospital-based Southern Chinese cohort, the cross-sectional analysis showed that, in Model 1 (without covariate adjustment), higher dairy intake quartiles were associated with lower odds of LMM. In particular, the OR for Quartile 4 was 0.25 (95% CI: 0.11–0.56) and was statistically significant. In Model 2 and Model 3, the association remained directionally consistent after adjustment for age, sex, BMI, smoking status, and hypertension, and higher dairy intake in Quartile 4 remained associated with lower odds of LMM (Table 5). Restricted cubic spline analysis further showed an inverse association between dairy intake and LMM, with a statistically significant overall association (P for overall < 0.01) (Figure 6).

**Table 5:** Association between dairy intake and LMM in the Southern Chinese cohort (*n* = 218; LMM = 107; non-LMM = 111).

Dairy intake category	Model 1 OR (95%CI)	*p*-value	Model 2 OR (95%CI)	*p*-value	Model 3 OR (95%CI)	*p*-value
Low dairy	Reference	–	Reference	–	Reference	–
High dairy	0.99 (0.98–0.99)	<0.01	0.99 (0.98–0.99)	<0.01	0.99 (0.98–0.99)	<0.01
Interquartile						
Quartile 1	Reference	–	Reference	–	Reference	–
Quartile 2	0.76 (0.35–1.66)	0.51	0.72 (0.33–1.60)	0.43	0.83 (0.36–1.91)	0.66
Quartile 3	0.33 (0.15–0.73)	<0.01	0.34 (0.15–0.74)	<0.01	0.34 (0.15–0.78)	0.01
Quartile 4	0.25 (0.11–0.56)	<0.01	0.23 (0.10–0.54)	<0.01	0.26 (0.11–0.61)	<0.01
P for trend		<0.01		<0.01		<0.01

Model 1 was unadjusted. Model 2 was adjusted for age and sex. Model 3 was adjusted for age, sex, BMI, smoking status, and hypertension. Only covariates actually collected in the Southern Chinese cohort were included in the adjusted models.

## 4 Discussion

This study primarily investigated the association between PHDI-US and LMM and then conducted a secondary exploratory analysis to examine whether a machine learning-informed refinement of the index might strengthen its observed association with LMM. The results showed that higher adherence to PHDI-US was associated with a lower risk of LMM. The PHDI-Dairy index, recalibrated via feature importance analysis, further strengthened this association. Stratified analyses indicated that the inverse association between PHDI-Dairy and low muscle mass was more pronounced in women, whereas no statistically significant association was observed in men. These findings suggest that the apparent inverse association of the dairy-upweighted dietary framework with LMM was most evident in women aged 46–60 years, and broader applicability across the full life course remains unproven. In this framework, the comparator indices served as contextual benchmarks, whereas the PHDI-Dairy analysis was intended as an exploratory extension rather than a co-primary objective.

In contrast to previous studies that have predominantly used sarcopenia as the primary endpoint, the present study conceptualizes LMM as an early, quantifiable phenotype along the sarcopenia spectrum. A large body of longitudinal studies and systematic reviews has demonstrated that, irrespective of concomitant declines in muscle strength, low muscle mass assessed by DXA or BIA is itself associated with increased risks of falls, fractures, functional limitations, and all-cause mortality (7, 35–37). This suggests that even before the full diagnostic criteria for sarcopenia are met, LMM per se already carries independent clinical prognostic significance (7). Taken together, these data support the use of LMM as a clinically meaningful intermediate phenotype along the sarcopenia spectrum. Against this background, increasing attention has shifted to modifiable upstream determinants of LMM, particularly overall lifestyle and dietary patterns, in order to identify strategies to preserve muscle mass earlier in the life course. For example, a recent NHANES-based cross-sectional study showed that higher Life’s Essential 8 scores—reflecting healthier dietary quality and lifestyle—were associated with lower odds of low muscle mass, supporting the link between overall healthy eating patterns and LMM (38).

An additional strength of this study is the inclusion of an independent Southern Chinese sample that provided supportive evidence for the inverse association between dairy intake and LMM. However, because the full PHDI-Dairy score was not reconstructed in that cohort and the dietary assessment method differed from that used in NHANES, this analysis should be interpreted as supportive evidence rather than formal external validation of the modified index. After increasing the dairy component weight, the modified index showed a stronger association with LMM in NHANES. The Southern Chinese cohort provided supportive evidence for the dairy-related finding.

In recent years, a growing body of research has focused on the relationship between overall diet quality and sarcopenia or its components, including LMM. Systematic reviews have shown that higher overall diet quality scores are generally associated with a lower risk of sarcopenia, as well as greater muscle mass, muscle strength, and physical performance. These scores typically include indices such as the Alternative Healthy Eating Index (AHEI), the Healthy Eating Index (HEI), the Mediterranean diet score, and composite scores reflecting adherence to dietary guidelines (39, 40). These indices capture diet quality from different perspectives and provide complementary evidence for how diet may influence LMM, a core measurable component on the pathway toward sarcopenia. As a comprehensive dietary scoring system, AHEI aims to assess the effects of diet on the risk of chronic diseases. A cross-sectional study including 300 older adults reported that higher Healthy Eating Index scores were associated with sarcopenia and its components, including low muscle mass (41). DII focuses specifically on evaluating the impact of diet on inflammation levels. Since chronic inflammation is closely related to the development and progression of sarcopenia, long-term adherence to a pro-inflammatory diet may activate inflammatory signalling pathways, promote muscle protein degradation, and inhibit muscle protein synthesis, thereby accelerating muscle loss and increasing the likelihood of developing sarcopenia (42). HEI primarily assesses diet alignment with dietary guidelines by scoring food groups and nutrient intake. Studies show depressive symptoms correlate negatively with muscle mass/strength, partly mediated by dietary energy intake (measured via HEI-2015), highlighting the need to optimise diet to reduce LMM risk in depressed middle-aged and older adults (43). A cross-sectional study found that adherence to the HEI-2015 may improve muscle strength in older adults (44).

Notably, muscle strength is only one component of sarcopenia, so its association with HEI does not imply a direct link between HEI and sarcopenia. MEDI has been proven to be a protective factor against diseases such as cardiovascular disease, diabetes, metabolic syndrome, and neurodegenerative diseases (45). Several studies have employed the Mediterranean diet score or its variants to investigate its relationship with muscle mass and sarcopenia-related characteristics. Overall, higher Mediterranean diet scores have been associated with greater relative muscle mass indices, such as skeletal muscle mass and appendicular lean mass, as well as a lower risk of sarcopenia and functional impairment (46–49). However, studies that have directly used LMM as a dichotomous outcome remain limited, and no significant associations have been observed in some Asian populations. The broader PHDI framework integrates human health and ecological sustainability, whereas the present study specifically evaluated its U.S. adaptation, PHDI-US. By advocating a plant-based diet with balanced plant protein intake and sustainable animal product consumption (33), PHDI reduces food system pressures and safeguards long-term food security, which is crucial for muscle health. As previous studies rarely considered both muscular health and ecological balance, PHDI offers novel perspectives for sarcopenia research and dietary intervention strategies.

Compared with the original PHDI-US, the dairy-upweighted score showed a stronger apparent inverse association within the derivation sample. Its magnitude was broadly comparable to that of AHEI, whereas HEI and MEDI were not statistically significant in the fully adjusted models. Thus, our results support only a limited comparison rather than a uniform ranking across all comparator indices. While the original EAT-Lancet report restricts dairy to reduce greenhouse gas emissions, our findings suggest that such restriction may be less optimal for maintaining muscle mass and preventing LMM in adults (19). We argue that the definition of “sustainability” for aging populations must encompass “physiological sustainability”-the ability to maintain functional independence. Multiple studies have demonstrated that, among middle-aged and older adults, increasing the intake of dairy products or whey protein-particularly when combined with resistance exercise-can stimulate muscle protein synthesis, enhance muscle strength, and improve muscle function and physical performance, although the effect sizes are generally small to moderate (50–53). Specifically, supplementation with 10 g of protein per day combined with low- to moderate-intensity exercise training has been shown to increase muscle mass in healthy older adults (54). Daily intake of 14–40 g of milk protein exerts a significant positive effect on appendicular muscle mass without altering handgrip or leg-press strength (50).

Notably, studies have shown that increasing protein intake (2 × 20 g/day) did not significantly improve muscle mass, strength, or functional performance in healthy, weight-stable older adults. Whether consuming > 20 grams of protein per meal is necessary for the maintenance of muscle mass and strength in older adults should be further explored in larger studies (55). Crucially, our refinement strategy maintains the core sustainability principle of the original EAT-Lancet framework by retaining the strict restriction on red meat intake. From an environmental perspective, red meat production is the most significant contributor to diet-related greenhouse gas emissions (56). In contrast, dairy products, while having a higher carbon footprint than plant-based foods, have a significantly lower environmental cost per gram of protein than red meat. The dairy-upweighted score may provide a hypothesis-generating example of how outcome-specific nutritional considerations could be explored within diet-quality indices derived from the planetary health framework. It avoids the heavy environmental burden associated with carnivorous diets while correcting the nutritional deficits of a strict plant-based diet for the aging demographic. However, environmental outcomes and long-term intervention effects were not assessed in this study; therefore, PHDI-Dairy should not be interpreted as a demonstrated “precision nutrition” or “green compromise” strategy.

Several biological pathways may plausibly link diet quality to muscle health, including protein quality, inflammatory regulation, and metabolic function, as suggested by prior human and preclinical studies (27, 37–40, 46, 48). However, because these pathways were not measured in our cross-sectional analysis, they should be considered contextual hypotheses rather than mechanistic explanations demonstrated by the present study. Existing mechanistic studies suggest that the occurrence of LMM/sarcopenia is the result of a combination of multiple factors. Chronic low-grade inflammation, insulin resistance, mitochondrial dysfunction, endocrine hormone changes, and reduced physical activity have created an internal environment in which skeletal muscle protein synthesis is inhibited and decomposed is hyperactive (57, 58). The dietary pattern advocated by PHDI, which incorporates high plant-based proteins (e.g., legumes, nuts) and moderate environment-friendly animal-sourced foods (e.g., grass-fed milk, deep-sea fish), can promote muscle anabolic metabolism through the abundant leucine present in high-quality proteins (40), and may also reduce muscle catabolism via anti-inflammatory effects associated with high fibre content in plants (59). The Omega-3 fatty acids in this diet can improve muscle mass and strength by mediating cell signalling and inflammation-related oxidative damage (60).

Additionally, previous studies have reported that dietary patterns and low protein intake may be associated with sarcopenia-related outcomes in older adults (61). Moreover, experimental evidence suggests that certain grain-derived bioactive compounds, such as alkylresorcinols, may help attenuate muscle atrophy by affecting energy metabolism (62). Animal and cell experiments have also suggested that certain grain-derived bioactives (e.g., alkyl resorcinol from wheat bran) may prevent muscle wasting by regulating energy substrate utilization and improving fatty acid metabolism disorders (62). Notably, A 4-month RCT showed that a high-protein diet with exercise and lean red meat increased muscle mass/strength (63), while a recent 24-week RCT found no such improvements versus the carbohydrate group (64). These contradictory results suggest that the true effect of red meat restriction on LMM needs further study and verification. Although PHDI-US is derived from the planetary health diet framework, the present study did not directly assess environmental footprints, carbon emissions, land use, water use, or other sustainability outcomes. Therefore, our findings should be interpreted as associations between a planetary-health-derived diet-quality index and LMM, rather than as direct evidence of combined human and planetary health benefits.

This study further investigated the differences in the association between PHDI-Dairy and LMM in different survey periods, gender and age subgroups through stratified analysis. The results showed that the protective effect of PHDI-Dairy was more significant in 2005–2012, mainly in women as a whole and in a subgroup of women aged 46–60 years. Stratification by survey year helps capture dynamic changes in diet and health behaviors over the life cycle, which is consistent with the conclusions of several prospective cohort studies focusing on “dietary trajectories.” Studies have shown that the trend of dietary quality from middle age to later life is closely related to the risk of subsequent death, body composition and physical function. For example, analysis by the China Health and Nutrition Survey (CHNS) suggests that there are significant differences between different long-term dietary quality trajectories and subsequent risk of all-cause mortality (65). A British birth cohort study reported that individuals who maintained higher diet quality during midlife exhibited more favorable body composition and less adiposity in later life (66). Studies conducted in U.S. and European populations have likewise shown that midlife dietary pattern trajectories are positively associated with late-life physical performance, including gait speed and handgrip strength (67). More recently, a Chinese cohort study demonstrated that maintaining consistently high or markedly improved diet quality from midlife to older age significantly reduces the risk of frailty in later life (68).

Against this background, we observed a stronger association between PHDI-Dairy and LMM in the earlier survey period (2005–2012). This discrepancy may be related to differences in baseline dietary patterns and exposure gradients across time periods, although the underlying reasons remain to be elucidated. The association was more evident in women, especially those aged 46–60 years; it was not significant in women aged 20–45 years. Evidence suggests that with advancing age, the muscle protein synthetic response to dietary protein and exercise stimuli progressively diminishes, a phenomenon referred to as “anabolic resistance” (69, 70). Systematic reviews and meta-analyses have shown that older adults with adequate protein intake have a lower risk of developing sarcopenia. However, among the very old, increasing protein intake alone often yields only limited improvements in muscle mass and strength, and needs to be combined with interventions such as resistance training to achieve more substantial benefits (71, 72). In the present study, the association between PHDI-Dairy and LMM was more pronounced in women, which is consistent with previous findings. Community-based studies have reported that women exhibit a higher prevalence of sarcopenia and a greater burden of related functional limitations than men, and that poor nutritional status contributes more heavily to their risk profile (73–75).

The stronger association observed in women, particularly those aged 46–60 years, may partly reflect unmeasured sex- and age-related factors, but these explanations remain hypothesis-generating. Menopause-related hormonal changes, including estradiol decline, may represent one possible biological hypothesis linking diet quality to muscle health in middle-aged women; however, menopausal status, circulating estrogen levels, and hormone therapy use were not directly measured in the present study (76–78). Moreover, middle-aged and older women are generally more likely than men to increase their intake of fruits, vegetables, whole grains, and low-fat dairy products, and tend to be more sensitive to changes in overall diet quality. Among women, persistent unhealthy dietary patterns are more strongly associated with the risk of developing frailty (79). Through stratified analyses, we can identify key determinants in different subpopulations and provide a more reliable basis for developing personalised PHDI-based dietary strategies to prevent LMM, thereby potentially delaying or reducing subsequent sarcopenic decline.

The strengths of this study include evaluating a diet-quality index derived from the planetary health framework in relation to LMM using nationally representative NHANES data. By optimising dietary indices using machine learning, dairy products were identified as key nutritional contributors, and a PHDI-Dairy index was developed, offering a new approach for dynamically refining nutritional indices to support muscle-mass preservation. Using large-scale NHANES data, we applied the multistage probability sampling design (with sample weights, stratification, and clustering), enhancing the population representativeness of our findings. Several limitations should be acknowledged. First, dietary indices were derived from processed NHANES recall data, but dietary measurement error and within-person variation remain possible. Second, the cross-sectional design precludes temporal inference. Third, the outcome was LMM rather than full sarcopenia. Fourth, PHDI-Dairy underwent internal cross-validation within NHANES, but the full composite score has not yet been externally evaluated in an independent cohort. Finally, the Southern Chinese analysis assessed dairy intake only and should be interpreted as supportive evidence for the dairy-related association rather than validation of the full modified index.

Although the Southern Chinese cohort improved the external contextual relevance of the dairy-related finding, it should be interpreted cautiously. The cohort differed from NHANES in sampling framework, dietary assessment method, and available covariates, and the full composite PHDI-Dairy score was not reconstructed in that population. Therefore, this analysis should be viewed as supportive cross-population evidence for the dairy component rather than as a formal external validation of the modified index itself. Importantly, this modified index was generated using a simplified exploratory reweighting strategy based on observed dairy intake and should not be interpreted as defining the optimal dairy intake level or the final component weighting structure. More rigorous optimization would require collaboration with nutrition scientists and statisticians, along with external validation in independent populations.

## 5 Conclusion

In this cross-sectional analysis of U.S. adults aged 20–60 years from NHANES, greater PHDI-US adherence was modestly associated with lower odds of LMM. An exploratory dairy-upweighted adaptation showed a stronger inverse association within NHANES, but this finding requires clearer methodological specification and external evaluation of the full composite score.

## Data Availability

Publicly available datasets were analyzed in this study. This data can be found here: The NHANES datasets analyzed in this study are publicly available from the National Center for Health Statistics. Data from the self-established Southern Chinese cohort are not publicly available due to ethical and privacy restrictions but may be made available from the corresponding author upon reasonable request and subject to institutional approval.

## Footnotes

1
https://www.cdc.gov/nchs/nhanes/index.htm

2
https://www.cdc.gov/nchs/

3
https://github.com/jamesjiadazhan/dietaryindex
