# Supplementary material for: Planetary health diet index and low muscle mass in adults aged 20–60 years: exploratory evaluation of a dairy-upweighted outcome-specific adaptation
Source: Front Nutr. 2026 Jul 15;13:1782327. doi: 10.3389/fnut.2026.1782327 (PMC13414832; doi:10.3389/fnut.2026.1782327)
Supplement: Supplementary file 1 [file Table_1.DOCX]

**Table S1**. Components and scoring criteria of the Planetary Health Diet Index for the United States (PHDI-US)

| **Scoring domain** | **Component** | **Score range** | **Intake range (%)*** | **Scoring direction** |
| --- | --- | --- | --- | --- |
| **Adequacy** | Nuts and Peanuts | 0–10 | 0.0 to ≥2.5 | Higher intake rewarded |
|  | Legumes | 0–10 | 0.0 to ≥8.5 | Higher intake rewarded |
|  | Fruits | 0–10 | 0.0 to ≥10.1 | Higher intake rewarded |
|  | Non-starchy Vegetables | 0–10 | 0.0 to ≥15.2 | Higher intake rewarded |
|  | Whole Grains | 0–10 | 0.0 to ≥25.0 | Higher intake rewarded |
| **Optimum** | Seafood and Substitutes | 0–10–0 | 0.0 → 1.4 → ≥5.1 | Highest score within target range |
|  | Tubers and Starchy Vegetables | 0–10–0 | 0.0 → 2.5 → ≥5.1 | Highest score within target range |
|  | Dairy | 0–10–0 | 0.0 → 12.6 → ≥25.3 | Highest score within target range |
|  | Unsaturated Oils | 0–10–0 | 0.0 → 2.0 → ≥4.0 | Highest score within target range |
| **Ratio** | DGV/total ratio | 0–5–0 | 0.0 → 33.3 → 100 | Highest score at target proportion |
|  | Rev/total ratio | 0–5–0 | 0.0 → 33.3 → 100 | Highest score at target proportion |
| **Moderation** | Eggs | 10–0 | ≤0.7 to ≥1.3 | Lower intake rewarded |
|  | Red and Processed Meat | 10–0 | ≤0.7 to ≥1.4 | Lower intake rewarded |
|  | Poultry | 10–0 | ≤1.5 to ≥2.9 | Lower intake rewarded |
|  | Saturated Fats | 10–0 | ≤0.3 to ≥0.6 | Lower intake rewarded |
|  | Added Sugars | 10–0 | ≤0.8 to ≥1.6 | Lower intake rewarded |

**Abbreviations**: DGV, dark green vegetables; Rev, red and orange vegetables; PHDI-US, Planetary Health Diet Index for the United States.

**Notes**: Score ranges: Adequacy, optimum, and moderation components are scored 0 to 10; ratio components are scored 0 to 5; Intake ranges are expressed as the percentage of total grams of foods and beverages consumed, excluding water; The index is structured into four domains (adequacy, optimum, ratio, and moderation) consistent with the original PHDI-US framework.

| Table S2 Stratified associations between the PHDI-dairy component score and sarcopenia | | | | | |
| --- | --- | --- | --- | --- | --- |
| Variable | OR | Lower | Upper | P value | P for interaction |
| **Gender** |  |  |  |  | 0.319 |
| Female | 0.99 | 0.97 | 1.01 | 0.399 |  |
| Male | 0.98 | 0.97 | 1 | 0.011 |  |
| **Age** |  |  |  |  | 0.031 |
| 20-44 | 0.97 | 0.96 | 0.99 | 0.002 |  |
| 45-59 | 1 | 0.98 | 1.02 | 0.966 |  |
| **Race** |  |  |  |  | 0.024 |
| Mexican American | 1 | 0.98 | 1.02 | 0.981 |  |
| Non-Hispanic Black | 0.97 | 0.93 | 1.02 | 0.309 |  |
| Non-Hispanic White | 0.97 | 0.95 | 0.99 | 0.013 |  |
| Other Hispanic | 0.97 | 0.94 | 1 | 0.09 |  |
| Other Race | 0.94 | 0.91 | 0.97 | <0.001 |  |
| **PIR** |  |  |  |  | 0.023 |
| <2 | 1 | 0.98 | 1.02 | 0.982 |  |
| >=2 | 0.97 | 0.96 | 0.99 | 0.002 |  |
| **Education** |  |  |  |  | 0.035 |
| Above high school | 0.97 | 0.96 | 0.99 | 0.002 |  |
| Below high school | 1 | 0.98 | 1.02 | 0.907 |  |
| High school | 1.01 | 0.98 | 1.03 | 0.599 |  |
| **BMI** |  |  |  |  | <0.001 |
| <18.5 | 1.03 | 0.85 | 1.25 | 0.774 |  |
| >=30 | 1.01 | 0.99 | 1.02 | 0.444 |  |
| 18.5-24.9 | 0.94 | 0.9 | 0.98 | 0.005 |  |
| 25-29.9 | 0.95 | 0.93 | 0.98 | <0.001 |  |
| **Smoking** |  |  |  |  | 0.26 |
| NO | 0.98 | 0.97 | 0.99 | 0.006 |  |
| YES | 0.99 | 0.97 | 1.01 | 0.532 |  |
| **Hypertension** |  |  |  |  | 0.205 |
| NO | 0.98 | 0.97 | 0.99 | 0.007 |  |
| YES | 1 | 0.98 | 1.02 | 0.848 |  |
| **Diabetes** |  |  |  |  | 0.41 |
| NO | 0.98 | 0.97 | 1 | 0.013 |  |
| YES | 1 | 0.97 | 1.02 | 0.723 |  |
| **Notes**: ORs and 95% CIs were estimated using stratified logistic regression. The P for interaction was used to assess subgroup differences. OR, odds ratio; CI, confidence interval; PHDI-US, Planetary Health Diet Index for the United States. | | | | | |
